# Environmental Orientation, Green Supply Chain Management, and Firm Performance: Empirical Evidence from Chinese Small and Medium-Sized Enterprises

**DOI:** 10.3390/ijerph17041199

**Published:** 2020-02-13

**Authors:** Xiangzhi Bu, Wilson V.T. Dang, Jianming Wang, Qiu Liu

**Affiliations:** 1 Department of Business Administration, Business School, Shantou University, Shantou, Guangdong 515063, China; xzpu@stu.edu.cn (X.B.); 19qliu1@stu.edu.cn (Q.L.); 2 Department of Business Administration, Dong Nai Technology University, Bien Hoa, Dong Nai 810000, Vietnam; wshdang1984@gmail.com; 3 School of Business Administration, Zhejiang University of Finance & Economics, Hangzhou 310018, China

**Keywords:** environmental orientation, green supply chain management, firm performance, Chinese SMEs

## Abstract

This study investigates the relationship between environmental orientation and firm performance with the mediating role of green supply chain management (GSCM). This study uses a survey questionnaire to collect data from 247 CEOs from Chinese small and medium-sized enterprises (SMEs). Structural equation modeling is used to analyze data and test hypotheses. Empirical results show that internal and external environmental orientations are positively related to the three elements of GSCM, namely, environmental selection, monitoring, and collaboration with suppliers which are also positively related to firm performance. In addition, results show that environmental selection, monitoring, and collaboration with suppliers mediates the relationship between internal and external environmental orientations and firm performance. The findings provide important implications for academic researchers and business managers in planning and implementing environmental strategies. In terms of theoretical implications, this study sheds a new light to current knowledge about the effect of environmental orientation on GSCM and firm performance of SMEs. This study also provides empirical evidence to clarify the mediating mechanism of GSCM in the link between environmental orientation and firm performance of SMEs. In terms of practical implications, this study provides knowledge for managers of SMEs to better understand the important role of environmental orientation and green supply chain management. Findings of this study provide knowledge for managers of SMEs to make their business policies better.

## 1. Introduction

Climate change and environmental deterioration cause public concerns for environmentally responsible behaviors [1]. Firms today are under increasing pressure from stakeholders and society to adopt an environmental orientation [2]. For example, customers demand more environment-friendly products, governments implement increased control and tightened regulations over business activities, and communities expect firms to protect and maintain green ecosystems. These pressures lead to a growing concern that firms should consider sustainability and environmental elements in their current production modes and business activities [3].

Given the importance of the above issues, firms may reluctantly engage in environmental activities [3] due to the need to balance economic and environmental performance [4]. Firms would unlikely adopt environmental involvement that increases costs and decreases performance. Consequently, firms only conform to regulations and transfer their environmentally irresponsible activities to other companies. For example, environmental standards, such as the United States’ Toxic Releases Inventory and Canada’s National Pollutants Release Inventory, require firms to reduce their environmental footprints. These regulations lead to several firms outsourcing their polluting activities [1]. Therefore, empirical evidence on how environmental orientation enhances firm performance would help businesses understand its importance and increase their willingness to invest in environmental activities. Several studies have investigated the issue of environmental orientation and its impact on firm performance. For example, Keszey [5] investigated the relationship between environmental orientation and firm performance with a sample of 296 firms in Hungary. Gigli, Landi, and Germani [6] illustrated an innovative technology for end-of-life vehicles fiber’s recycling from an environmental point of view. Landi et al. [7] conducted a research of comparative evaluation of the environmental impacts related to three different end of life scenarios for the textile fibers. Similarly, Gigli, Landi, and Germani [8] also used mathematical model to illustrate the impact of environment. However, only the bivariate relationship of environmental orientation with firm performance is extensively studied [9,10,11], whereas its influence on green supply chain management (GSCM) that in turn affects firm performance remains largely unexplored.

Stakeholder theories posit that legitimacy and resources are the two main factors driving firms to increase their environmental orientation [2]. Moreover, to fulfill expectations of public society, government institutions, and customers, firms should assess and improve the environmental performance of their suppliers. Essentially, GSCM helps firms create capabilities to address pressures from stakeholders while establishing a competitive advantage to enhance firm performance [1]. In this study, we investigate the influence of environmental orientation on GSCM, which in turn affects firm performance. Following institutional and stakeholder theories, we argue that legitimacy and resources are the two major factors driving firms to manage, monitor, and collaborate with suppliers to enhance their green capability. In addition, according to resource-based view (RBV), we infer that GSCM helps firms create increasing values for customers and construct a competitive advantage, which enhances firm performance.

China is now the second largest economy and the largest developing market in the world. Majority of the world’s manufacturing are carried out in China [10]. Economic development and industrialization are accompanied with substantial environmental pollution [11], which has gained focal attention in Chinese society. As a result, the Chinese government and firms have increased their efforts to minimize the impact of business activities on the environment. For instance, the Chinese government has developed policies and regulations that require firms to reduce environmental harms caused by their operations [12]. Business firms have also increased their efforts to implement ISO 14001 and recently GSCM [13]. GSCM contributes to the improvement of environmental performance via reduction of pollution and energy consumption while also increasing economic benefits [13]. The value of GSCM is particularly salient for Chinese firms, where environmental performance is increasing in importance in the business industry [12].

Recent decades have witnessed a dramatic rise of not only giant firms but also of small and medium-sized enterprises (SMEs) in China. Toward the end of 2013, China has approximately 97.33% registered SMEs that contribute approximately 60% of China’s GDP [14]. Such contribution is particularly vital to economic development, and SMEs cannot be ignored in determining environmental issues in China’s context [11]. Furthermore, Chinese SMEs often operate in a market with strong competitive and institutional forces. Compared with large firms, SMEs are smaller in size and possess limited resources and advantages [15]. Challenges of resource scarcity and competitiveness require SMEs to seek methods to secure their legitimacy and to be more flexible to compete with leading firms in the industry [16]. As environmental issues cause rising pressure for business firms, SMEs should adopt environmental orientation and utilize GSCM to create their competitive advantage. These two factors can help SMEs obtain legitimacy and resources while enhancing their competitive capability and firm performance. Therefore, this study considers Chinese SMEs to analyze how environmental orientation affects GSCM, which in turn influences firm performance.

The remainder of this paper is structured as follows. The next section reviews relevant previous literature and develops hypotheses. The third section describes the methodology and the fourth section shows empirical results. Conclusions and suggestions for future research are provided in the final section. 

## 2. Literature Review and Hypotheses Development

### 2.1. Environmental Orientation

Environmental orientation is defined as “the extent to which a company integrates ecological issues into its business strategy to reduce the harmful effects of its business-related activities on the natural environment” (p. 22) [17]. Environmental orientation is a core concept in the study of environmental management [2] and indicates the recognition and integration of environmental concerns into firms’ operation to manage environmental issues. The purpose is to minimize the impact a firm produces on the environment [18]. Banerjee [18] divided environmental orientation into external, referring to how firms respond and fulfill the expectations of external stakeholders on environmental issues, and internal, which refers to firms’ internal values, ethical standards, and commitment to protect the environment [2].

### 2.2. Environmental Orientation and GSCM

Stakeholder theory indicates the interdependence between business firms and their stakeholders. Firms have to manage different pressures from various vital institutions [19]. For example, government agencies propose new regulations and standards to reduce firms’ negative environmental impact, communities hold higher expectations for firms’ citizenship behavior, and customers demand environment-friendly products [2]. To face these pressures from stakeholders, firms implement specific strategies such as pollution prevention, product stewardship, and clean technology [20]. Dincer [21] indicated that renewable energy technologies and efficient energy utilization are effective solutions for environmental problems. Florida and Davidson [22] emphasized the importance of environmental management system to fulfill business goals and enhance environmental performance. Kjaerheim [23] suggested that cleaner production is an effective strategy to obtain environmentally sustainable objectives. Environmental strategies are also extended to the supply chain management [3,12,15,24,25]. From this perspective, firms may be more inclined to engage in GSCM because of the strong need to respond to environmental demands from stakeholders. Strategies that focus on GSCM help firms to obtain support, legitimacy, and resources from stakeholders. 

Furthermore, according to stakeholder theory, environmental issues have become a key concern for government agencies, decision makers, and business firms in today’s business environment. Business firms have to respond to pressure from various stakeholders in dealing with environmental problems [26]. One possible strategy for business firms is to commit to environmental issues by focusing on either internal or external organization [27]. From external organization perspective, suppliers play an important role in building capability and competitive advantage for firms because they provide key materials and components for firms [28]. In order for firms to fulfill external stakeholders’ expectations, firms may seek and collaborate with different suppliers to solve environmental issues [29]. For example, firms can partner with suppliers to develop new green materials and components, engage in green R&D activities, or build green policy and management systems [30]. By integrating environmental protection into supply chain management, firms can develop green capability and build favorable green reputation in the eyes of stakeholders [31]. In other words, to respond to external stakeholders for environmental protection, firms can integrate environmental activities into their supply chain management. By seeking, monitoring, and collaborating with suppliers, firms can develop green capability and secure green resources. Consequently, firms can effectively obtain supports from external stakeholders [32]. Thus, external environmental orientation may exert a positive influence on GSCM. The following hypotheses are developed.

**H1a.**  *External environmental orientation positively affects environmental selection of suppliers.*

**H1b.**  *External environmental orientation positively affects environmental monitoring of suppliers.*

**H1c.**  *External environmental orientation positively affects environmental collaboration with suppliers.*

In a highly complex and competitive market, consumers have multiple buying options. As consumers’ purchase power increases, they demand higher quality and healthier products for their money. Thus, environmental businesses gradually grow into good business [33]. Albino, Balice, and Dangelico [34] suggested firms to implement environmental strategies to satisfy customers’ needs. Designing green products that minimize its environmental impacts during its entire lifecycle is an example [35]. Bakker, Fissher, and Brack [36] suggested that going “green” requires firms to address their environmental efforts early in the supply chain. Environmental sustainability is becoming increasingly common in corporate culture [34]. Furthermore, corporate leaders often initiate environmental orientation. From these leaders’ perceptions, attitudes, and behaviors, environmental ideology may spread throughout the entire firm and become core cultural values and perceptions. Consequently, internal firm members seek to establish environmental businesses and minimize the environmental impacts of their operations [35]. Chiefly, to provide environmental-friendly products for customers, corporate leaders may develop environmental strategies that shape the collective consciousness regarding the importance of environmental issues. Such core cultural values and beliefs may motivate firms to exert their internal efforts toward GSCM.

According to stakeholder theory, consumers are one of the most important stakeholders of a firm [26]. Because today’s consumers demand more environmentally friendly products. Business firms have to produce more green products to satisfy consumers’ demands [27]. Except focusing on external organization, integrating environmental issues into internal organization is another effective environmental strategy [15]. For example, firms can invest to build green facilities and equipment, engage in green R&D activities, build green organizational culture, etc. [30]. Building green capability requires firms combine internal and external resources because internal activities are closely related to external activities [37]. Therefore, to build firms’ green capability and produce green products for customers, firms’ internal operations have to depend largely on external activities with suppliers. Furthermore, supply chain management and internal operation is often combined together in a firm’s value-chain. As these activities linked together, when firms engage in environmental activities and integrate environmental issues into internal organization, firms have to combine it with external suppliers [38]. For example, when a firm invests in green R&D activities or uses green facilities and equipment to produce green products, they have to seek and build relationships with green suppliers because firms need green materials and resources from these suppliers [28]. Therefore, it is argued that when firms integrate environmental issues into internal organization, they have to seek, monitor, and collaborate with green suppliers because firms’ internal activities are closely related to their supply chain management. Thus, the following hypotheses are developed.

**H2a.**  *Internal environmental orientation positively affects environmental selection of suppliers.*

**H2b.**  *Internal environmental orientation positively affects environmental monitoring of suppliers.*

**H2c.**  *Internal environmental orientation positively affects environmental collaboration with suppliers.*

### 2.3. GSCM and Firm Performance

GSCM is an effective environmental strategy for firms to gain competitive advantage and enhance firm performance [39]. According to RBV, firms acquire sustainable competitive advantage when they obtain resources that are valuable, scarce, inimitable, and non-substitutable [40,41]. These strategic resources can be constructed through GSCM [36]. With the increasing demand for green products and higher government and societal concerns for the environment, firms should seek and manage relationships with green suppliers to produce safer and less costly products. Such management can help firms obtain and secure sustainable materials that are useful for producing green products [34]. By collaboration and integration with green suppliers, firms likewise develop unique capabilities in operational process, R&D activity, and product development [3]. GSCM is proven to enhance corporate performance. For example, Hanna, Newman, and Johnson [42] found a positive relationship between environmental management systems and operational performance. Zhu et al. [12] indicated that GSCM has a progressive impact on operational performance, while Green et al. [3] demonstrated its positive relation to firm performance. Chan [43] also suggested that GSCM helps firms reduce legal risks associated with environmental violation, improve corporate reputation, and enhance abilities to satisfy the demand of environmentally conscious customers. Consequently, GSCM improves firm performance.

In sum, according to RBV, GSCM helps firms to build green capability that produces green products to satisfy customers’ needs [27]. This is because when seeking, monitoring, and collaborating with suppliers, firms can obtain green materials and components and secure green resources from suppliers [28]. In other words, by partnering with suppliers, firms can develop green R&D activities, which help firms produce environmentally friendly products to meet customers’ demands [30]. Consequently, firms’ performance will be enhanced as a result of this capability [26]. Therefore, the following hypotheses are developed.

**H3.**  *Environmental selection of suppliers positively affects firm performance.*

**H4.**  *Environmental monitoring of suppliers positively affects firm performance.*

**H5.**  *Environmental collaboration with suppliers positively affects firm performance.*

### 2.4. The Mediating Role of GSCM

Legitimacy and resources are critically important for the survival and development of any firm [39] and obtaining them requires fulfilling the expectations of numerous institutions in the marketplace. By incorporating environmental strategy into firms’ strategic planning, firms can secure support from their stakeholders [34]. One of the most effective means to be environmentally oriented is by extending sustainable strategies to the supply chain [44,45]. GSCM not only helps firms effectively respond to stakeholders’ expectations, but also builds good reputation and develops green capability (e.g., clean production, non-polluting, and minimal waste production process, green R&D, and green products) that creates competitive advantage and enhances firm performance [3,25]. Therefore, environmental orientation requires firms to invest increased efforts and resources into GSCM, which in turn creates green capability and competitive advantage. Consequently, firms achieve superior performance from such environmental strategies.

In sum, according to stakeholder theory, different actors in society expect firms to commit to environmental protection [26]. To respond to this pressure, firms can integrate environmental issues into their business strategy by either focusing on internal or external organization [27]. From internal organization perspective, firms can invest to build green capability in operation and management systems (e.g., building green facilities and equipment, engaging in green R&D activities, and developing green policy and management systems) [30]. From external organization perspective, firms can also build a green image and positive reputation with external stakeholders (e.g., support community in environmental protection or engage in citizenship behaviors) [27]. Both internal and external activities will enhance firms to seek, monitor, and collaborate with suppliers to build green capability because firms have to combine internal operation with external activities with suppliers [38]. Specifically, firms will obtain green materials and components and secure green resources from suppliers which are important factors in building firms’ green capability [37]. Furthermore, according to RBV, when firms build good relationships with suppliers, they can obtain and develop strategic resources from suppliers [28]. This will help firms develop and build green capability which helps firms produce more environmentally products to meet customers’ expectations [27,30]. Consequently, firms will sell more green products and earn more performance [26]. Therefore, it is expected that when firms engage in environmental orientation, they will seek to collaborate and partner with suppliers to build green capability. Firms will produce more environmentally friendly products to satisfy customers’ needs. As a result, firm performance will be increased. Thus, the following hypotheses are developed.

**H6a.**  *Environmental selection of suppliers mediates the relationship between external environmental orientation and firm performance.*

**H6b.**  *Environmental monitoring of suppliers mediates the relationship between external environmental orientation and firm performance.*

**H6c.**  *Environmental collaboration with suppliers mediates the relationship between external environmental orientation and firm performance.*

**H6d.**  *Environmental selection of suppliers mediates the relationship between internal environmental orientation and firm performance.*

**H6e.**  *Environmental monitoring of suppliers mediates the relationship between internal environmental orientation and firm performance.*

**H6f.**  *Environmental collaboration with suppliers mediates the relationship between internal environmental orientation and firm performance.*

**Figure 1:**
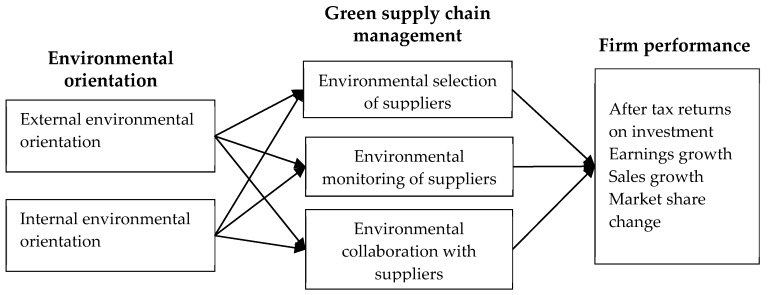
Study framework.

Figure 1 shows the study framework and relationships between variables in this study.

## 3. Methodology

### 3.1. Sample and Data Collection

This study used a survey methodology. The questionnaire was designed and translated from English into Chinese and then back to English using backward translation by two experts who are proficient in both languages. To ensure clarity and define each measurement item, we conducted a pre-test with 20 managers in China. Subsequently, we invited different Chinese SMEs to complete the revised questionnaire. A total of 480 CEOs voluntarily participated in the survey and completed the Chinese version. The survey was conducted from February to April in 2019. After eliminating invalid samples with missing data, 247 valid questionnaires were obtained for the final sample, yielding a response rate of 51.46%.

Table 1 presents the demographic profiles of the respondents, as follows: 49.8% were male and 50.2% were female; 83.0% held college or university degrees, 15.4% had graduate degrees or above, and only 1.6% had a high school diploma or under; Approximately 65.6% were 31–35 years old, 28.3% were 26–30 years old, and only 6.1% were 36 years or older; Approximately 30.0% of respondents worked in the manufacturing industry, 28.7% worked in the real estate industry, 23.5% worked in the finance industry, 4% worked in the electronics and telecommunications industry, and the remaining worked in other industries.

### 3.2. Measures

All constructs and items were adopted from previous studies. Table 2 displays the constructs and item measurements. Environmental orientation was measured using eight items from Chan et al. [2], with four items each for internal environmental orientation (1–4) and external environmental orientation (5–8). GSCM was measured using nine items from Gavronski et al. [1]. Environmental selection, monitoring, and collaboration were each measured with three items (items 9–11, 12–14, and 15–17, respectively). Firm performance was measured using four items (18–21) adopted from Chan et al. [2]. The value of each construct was calculated by averaging all values of its items. 

## 4. Results

### 4.1. Descriptive Statistics

Table 3 presents the means, standard deviations, and Pearson correlations among variables in this study. The results show that internal environmental orientation was significantly and positively related to environmental selection of suppliers (r = 0.16, *p* < 0.01), environmental monitoring of suppliers (r = 0.05, *p* < 0.01), and environmental collaboration with suppliers (r = 0.15, *p* < 0.01). External environmental orientation was also significantly and positively related to environmental selection of suppliers (r = 0.22, *p* < 0.01), environmental monitoring of suppliers (r = 0.19, *p* < 0.01), and environmental collaboration with suppliers (r = 0.18, *p* < 0.01). Furthermore, environmental selection, monitoring, and collaboration with suppliers were significantly and positively related to firm performance at (r = 0.46, *p* < 0.01), (r = 0.35, *p* < 0.01), and (r = 0.06, *p* < 0.01), respectively.

### 4.2. Measurement Model

Following Kline [46], confirmatory factor analysis (CFA) was conducted with AMOS 18 statistical software. The hypothesized model reveals satisfactory goodness-of-fit with the data when χ^2^/d.f. ratio is less than 3, GFI is greater than 0.90, CFI is greater than 0.90, NFI is greater than 0.90, TLI is greater than 0.90, and RMSEA is less than 0.08. Table 4 exhibits that all criteria satisfy the benchmark fit indices. Thus, the conceptual model fits the data reasonably well.

To test the convergent and discriminant validity, we determine the composite reliability (CR) and average variance extracted (AVE). According to Hair et al. [47], convergent validity is satisfied if CR value is greater than 0.70 and AVE value is greater than 0.50. Table 5 shows that the CR and AVE values of all constructs meet these requirements. Therefore, convergent validity is satisfactory in this study. Furthermore, Hair et al. [47] stated that discriminant validity is satisfied if the square root of AVE value is greater than the off-diagonal elements in the corresponding rows and columns of the Pearson correlation matrix. As displayed in Table 3, the square roots of AVE on the main diagonal are greater than those of the off-diagonal elements in the corresponding rows and columns of the matrix. Thus, discriminant validity is also satisfactory.

To test the internal consistency reliability, we calculate for Cronbach’s alpha [47]. Table 5 shows that Cronbach’s alpha for internal and external environmental orientations, environmental selection, monitoring, and collaboration with suppliers, and firm performance were 0.94, 0.88, 0.93, 0.92, 0.96, and 0.94, respectively. These values are all above the suggested criteria of 0.60 [47]. Thus, the results indicate good reliability of the measurement scales.

To detect the possible problem of common method bias, we adopt the proposed test by Podsakoff et al. [48]. Common method bias may arise if the results of principal component analysis indicate a single factor or a first factor explains the majority of the variance from unrotated factor solution. The results show six factors with an eigenvalue above 1.0, which accounts for 73.96% of variance. The first factor accounted for 12.08% of variance. In addition, CFA of one-factor model is conducted to confirm the problem of common method bias. The results show a poor model fit (χ^2^/df = 11.41, GFI = 0.50, CFI = 0.66, NFI = 0.64, TLI = 0.63, and RMSEA = 0.21). Thus, common method variance may be not a serious problem in this study.

### 4.3. Structural Model

Hypotheses are tested using structural equation model (SEM), an advanced statistical method that manages causal relationships among several variables in a single research framework. SEM has several advantages in comparison with multiple regression analysis. For example, SEM deals with latent and observed variables. The causal relationships among variables in the same model are also addressed to allow control over errors (dealing with several independent and dependent variables in a single model). SEM is a family of related statistical methods that combine CFA, path analysis, and regression analysis. 

**Figure 2:**
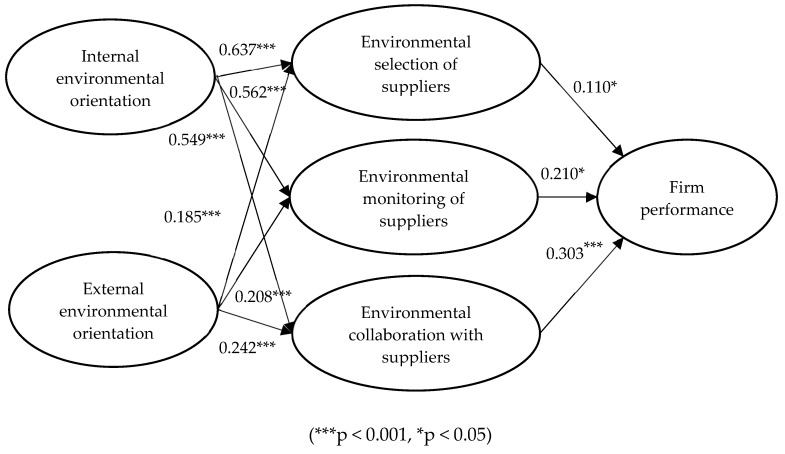
Hypotheses testing results.

Figure 2 presents the results of the structural model. Internal environmental orientation is significantly and positively related to environmental selection of suppliers (β = 0.637, *p* < 0.001), environmental monitoring of suppliers (β = 0.562, *p* < 0.001), and environmental collaboration with suppliers (β = 0.549, *p* < 0.001). Thus, H1a, H1b, and H1c are supported. External environmental orientation is also significantly and positively related to environmental selection of suppliers (β = 0.185, *p* < 0.001), environmental monitoring of suppliers (β = 0.208, *p* < 0.001), and environmental collaboration with suppliers (β = 0.242, *p* < 0.001), thereby supporting H2a, H2b, and H2c. Furthermore, environmental selection of suppliers is significantly and positively related to firm performance (β = 0.110, *p* < 0.05), which supports H3. Environmental monitoring of suppliers is significantly and positively related to firm performance (β = 0.210, *p* < 0.05), supporting H4. Finally, environmental collaboration with suppliers is significantly and positively related to firm performance (β = 0.303, *p* < 0.001), providing support for H5.

To test the mediating effect of GSCM between environmental orientation and firm performance, we follow the method proposed by Preacher, Rucker, and Hayes [49]. A bootstrap analysis is conducted with 5000 bootstrap samples. First, we test the indirect effect of external environmental orientation on firm performance through GSCM. This indirect effect is statistically significant (external environmental orientation→environmental selection of suppliers→firm performance: β = 0.232, *p* < 0.001, 95% CI = [0.137, 0.328]; external environmental orientation→environmental monitoring of suppliers→firm performance: β = 0.212, *p* < 0.001, 95% CI = [0.111, 0.327]; external environmental orientation→environmental collaboration with suppliers→firm performance: β = 0.194, *p* < 0.001, 95% CI = [0.081, 0.336]). Thus, H6a, H6b, and H6c are supported. Furthermore, the indirect effect of internal environmental orientation on firm performance is tested through GSCM. This indirect effect is statistically significant (internal environmental orientation→environmental selection of suppliers→firm performance: β = 0.177, *p* < 0.001, 95% CI = [0.062, 0.296]; internal environmental orientation→environmental monitoring of suppliers→firm performance: β = 0.166, P < 0.001, 95% CI = [0.063, 0.294]; internal environmental orientation→environmental collaboration with suppliers→firm performance: β = 0.126, *p* < 0.001, 95% CI = [0.019, 0.265]). Thus, H6d, H6e, and H6f are supported. 

To further confirm the mediating effect of GSCM in the link between environmental orientation and firm performance, we followed Moslehpour et al. [50], Moslehpour et al. [51], and Moslehpour et al. [52] to conduct Table 6 to show the direct, indirect, and total effects of environmental orientation on firm performance through GSCM. 

As indicated in Table 6, external environmental orientation has a significant direct and indirect effect on firm performance through environmental selection, monitoring, and collaboration with suppliers. The total effect of external environmental orientation on firm performance was also statistically significant. These results show that environmental selection, monitoring, and collaboration with suppliers partially mediate the relationship between external environmental orientation and firm performance. Therefore, hypotheses H6a, H6b, and H6c were supported.

Furthermore, results in Table 6 also show that internal environmental orientation has a significant direct and indirect effect on firm performance through environmental selection, monitoring, and collaboration with suppliers. The total effect of internal environmental orientation on firm performance was also statistically significant. These results show that environmental selection, monitoring, and collaboration with suppliers partially mediate the relationship between internal environmental orientation and firm performance. Therefore, hypotheses H6d, H6e, and H6f were supported. Table 7 summarized the results of all hypotheses testing in this study.

## 5. Discussion

This study investigates the relationship between environmental orientation and firm performance with the mediating role of GSCM in Chinese SMEs. The results show that internal environmental orientation is positively related to environmental selection, monitoring, and collaboration with suppliers. This result implies that 

Furthermore, external environmental orientation is positively related to environmental selection, monitoring, and collaboration with suppliers. In addition, the three elements of GSCM (environmental selection of suppliers, environmental monitoring of suppliers, and environmental collaboration with suppliers) are also positively related to firm performance. Ultimately, GSCM is found to have a mediating effect between the relationship between environmental orientation and firm performance of Chinese SMEs. The findings of this research offer novel and important implications for academic researchers and business managers regarding environmental issues.

### 5.1. Theoretical Implications

First, previous studies only focus on the direct relationship between environmental orientation and firm performance [5]. The mediating role of GSCM in this relationship remains largely unexplored in literature. This creates a lack of understanding on the mediating mechanism into this relationship. To fill this research gap, the present study determines the effects of external and internal environmental orientation on GSCM, including environmental selection, monitoring, and collaboration with suppliers, which in turn influences firm performance. Findings of this study sheds a new light on the mediating role of GSCM in the relationship between environmental orientation and firm performance. Our findings help to extend current knowledge on the indirect effect of environmental orientation on firm performance through GSCM. Therefore, this study provides implications for researchers who may have interest to study the relationships between environmental issues, green supply chain, and firm performance.

Second, this study reveals a positive effect of external environmental orientation on GSCM. This result indicates that environmental strategy focusing on GSCM is an effective means for firms to respond to pressures from various external stakeholders (e.g., government agencies, communities, and customers) [3] and even obtain legitimacy, support, and resources [12,15,24]. Specifically, in order for firms to fulfill external stakeholders’ expectations, firms may seek and collaborate with different suppliers to solve environmental issues [29]. Suppliers play an important role in building capability and competitive advantage for firms because they provide key materials and components for firms [28]. By integrating environmental protection into supply chain management, firms can develop green capability and build favorable green reputation in the eyes of stakeholders [31]. Consequently, firms can effectively obtain supports from external stakeholders [32]. Our findings support the notions that firms can effectively respond to pressure from external stakeholders by partnering with suppliers to solve environmental issues [27]. Similarly, this study finds that internal environmental orientation is also positively related to GSCM. This result implies that environmental ideology initiated by corporate leaders may become corporate culture, values, and beliefs of a firm [34]. The core cultural values and beliefs may motivate internal firms to actively engage in GSCM [35]. In other words, building green capability requires firms combine internal and external resources because internal activities are closely related to external activities [37]. To build firms’ green capability and produce green products for customers, firms’ internal operations have to depend largely on external activities with suppliers. When firms integrate environmental issues into internal organization, they have to seek, monitor, and collaborate with green suppliers because firms need green materials and resources from these suppliers [28]. Our findings enrich knowledge about the influence of internal and external environmental orientation on GSCM. Therefore, this study extends stakeholder theory and uses it as a foundation to combine firms’ environmental orientation with GSCM. Our findings may provide implications for researchers who may be interested in studying the integration of environmental protection into GSCM. 

Third, GSCM is found to have a positive effect on firm performance. This result presents that environmental strategy focusing on GSCM may motivate firms to select, collaborate, and integrate with suppliers [25]. Such strategy helps firms develop a unique “green” capability in the operational process, R&D activity, and product development [3]. Consequently, firms are able to satisfy the demand of environmentally conscious customers and gain superior performance [43]. Our finding is consistent with Noh and Kim [28] which stated that when partnering with suppliers, firms can develop green R&D capability, which helps firms produce environmentally friendly products to meet customers’ demands. Firms’ performance will be enhanced as a result of this capability. Therefore, this study provides further empirical evidence on the relationship between GSCM and firm performance which enriches knowledge in current literature.

Finally, this study utilizes Chinese SMEs as target of the investigation to analyze how the effect of environmental orientation on GSCM, which in turn affects firm performance. To our knowledge, environmental concerns of Chinese SMEs are unexplored. Environmental pollution has been a severe problem in China recent years. As pressure from various stakeholders increases, small and medium sized firms in China have to integrate environmental issues into their business strategy. Therefore, this study contributes to our knowledge of the effect of environmental orientation in Chinese SMEs. Our findings provide initial evidence for researchers who will study the issue of environmental protection and GSCM for SMEs in Chinese context.

### 5.2. Practical Implications

This study also provides implications for business managers. Environmental issues are becoming a public concern in today’s business industry. Pressures from various stakeholders force firms to engage in environmental protection activities. Thus, it is suggested that firms should integrate environmental issues into their strategic planning. Environmental strategies that focus on GSCM help firms secure support and resources and respond effectively to pressures from stakeholders. Furthermore, firms should extend their environmental strategies to the supply chain because GSCM helps firms develop unique capability that leads to competitive advantage and improve firm performance. For example, GSCM may enhance cleaner production, develop green R&D capability, produce environmental-friendly products that satisfy customers’ need, and create sustainable competitiveness through environmental orientation.

This study provides special implications for business managers in Chinese SMEs. Based on findings in this study, it is suggested that Chinese managers should invest more to engage in environmental activities. They should commit to both internal and external environmental protection because environmental issues are major concerns of different stakeholders in society in today’s business environment. Furthermore, it is suggested that business managers in Chinese SMEs should seek, collaborate, and partner with key suppliers to deal with environmental problems. For example, business managers should plan some partnership programs with suppliers to develop green materials and components, engage in green R&D activity to develop green products, and change production systems that reduce impacts on environment. Building green capability may be a competitive advantage for Chinese SMEs because environmental protection is a key tendency in today’s business environment. Altogether, it is suggested that business managers in Chinese SMEs should actively engage in environmental protection by integrating environmental issues into their business activities and supply chain management. Doing so will create green competitive advantage and increase firm performance for Chinese SMEs.

### 5.3. Limitations and Future Research

Several limitations are acknowledged in this study. First, the sample data are collected solely from Chinese SMEs, and thus the findings may limit the generalization of this study. Data from other emerging countries (e.g., India, Southeast Asia, Russia, or Brazil) can be collected to provide further evidence of the effects of environmental orientation in developing markets. Second, cross-sectional data are widely used in psychological and organizational research. Although causal relationships between variables can be tested using cross-sectional data, the results may be biased. Therefore, future research can collect longitudinal data to observe the long-term effects of environmental orientation on GSCM and firm performance. Third, the self-report survey may lead to biased results due to the same respondents providing measures for the dependent and independent variables simultaneously. Future research should use objective and less-biased method to collect survey data. Finally, this study only focuses on three aspects of GSCM, namely, environmental selection, monitoring, and collaboration with suppliers. Future research can address different dimensions of GSCM, such as green information systems, green purchasing, and eco-design.

## 6. Conclusions

This study makes an important contribution to the literature by presenting and testing a model that investigates the impact of environmental orientation on firm performance with the mediating role of GSCM for SMEs in China. Findings confirm the positive relationship between environmental orientation and GSCM and that between GSCM and firm performance. The positive mediating role of GSCM is also confirmed in the relationship between environmental orientation and firm performance. Thus, this study extends knowledge about the impact of environmental orientation in the context of Asian emerging markets.

## Figures and Tables

**Table 1:** Demographic profile.

Variable	Frequency	Percentage (%)
Gender		
Male	123	49.80%
Female	124	50.20%
Education		
High school or below	4	1.60%
College or university	205	83.00%
Graduate or above	38	15.40%
Age		
Under 20	0	0.00%
21–25	0	0.00%
26–30	70	28.30%
31–35	162	65.60%
36 or above	15	6.10%
Industry		
Manufacturing	74	30.00%
Mining	1	0.40%
Construction	7	2.80%
Electricity and gas	9	3.60%
Logistics	1	0.40%
Finance	58	23.50%
Electronics and telecommunications	10	4.00%
Wholesale and retail	5	2.00%
Restaurant	4	1.60%
Tourism	7	2.80%
Real estate	71	28.70%
N = 247		

**Table 2:** Constructs and item measurements.

Constructs	Items	Sources
Internal environmental orientation	(1 = strongly disagree, 2 = disagree, 3 = neutral, 4 = agree, 5 = strongly agree) 1. Our firm exerts concerted efforts to allow each employee to understand the importance of environmental preservation.	[2]
	2. Our firm has clear policy statements urging environmental awareness in all areas of operation.	
	3. Environmental preservation is highly valued by our firm members.	
	4. Environmental preservation is a central corporate value of our firm.	
External environmental orientation	5. The developments in the natural environment affect our firm’s business activities.	[2]
	6. The financial well-being of our firm depends on the state of the natural environment.	
	7. Environmental preservation is vital to our firm’s survival.	
	8. Various external stakeholders expect our firm to preserve the environment.	
Environmental selection of suppliers	During the past three years, to what extent did your plant engage in the following environmental activities with your potential primary suppliers? (1 = not at all, 2 = somewhat, 3 = moderately, 4 = quite a bit, 5 = great extent).	[1]
	9. Request potential primary suppliers to provide evidence of all environmental licenses and permits.	
	10. Require potential primary suppliers to have an implemented environmental management system (e.g., ISO 14001).	
	11. Have environmental specialists audit potential primary suppliers’ plants.	
Environmental monitoring of suppliers	During the past three years, to what extent did your plant engage in the following environmental activities with your existing primary suppliers (i.e., the 155–20% most important suppliers)? (1 = not at all, 2 = somewhat, 3 = moderately, 4 = quite a bit, 5 = great extent).	[1]
	12. Send environmental questionnaires to existing primary suppliers to monitor their compliance.	
	13. Ask existing primary suppliers to commit to waste reduction goals.	
	14. Have environmental criteria in periodic evaluation of existing primary suppliers.	
Environmental collaboration with suppliers	During the past three years, to what extent did your plant engage in the following environmental activities with your existing primary suppliers (i.e., the 15–20% most important suppliers)? (1 = not at all, 2 = somewhat, 3 = moderately, 4 = quite a bit, 5 = great extent).	[1]
	15. Work together to reduce environmental impact of our activities.	
	16. Conduct joint planning to anticipate and resolve environment-related problems.	
	17. Make joint decisions about methods to reduce overall environmental impact of our products.	
Firm performance	During the past three years, rate your firm’s performance relative to your competitors (1= much worse, 5 = much better)	[2]
	18. After tax returns on investment	
	19. Earnings growth	
	20. Sales growth	
	21. Market share change	

**Table 3:** Means, standard deviations, and Pearson correlations.

Constructs	Mean	S.D.	1	2	3	4	5	6
1. Internal environmental orientation	3.39	1.05	0.89					
2. External environmental orientation	3.20	1.07	0.18 **	0.81				
3. Environmental selection of suppliers	3.33	1.25	0.16 **	0.22 **	0.91			
4. Environmental monitoring of suppliers	3.15	1.22	0.05 **	0.19 **	0.27 **	0.90		
5. Environmental collaboration with suppliers	3.21	1.22	0.15 **	0.18 **	0.23 **	0.29 **	0.97	
6. Firm performance	3.56	0.97	0.17 **	0.43 **	0.46 **	0.35 **	0.06 **	0.90

Note: n = 247, ** *p* < 0.01, square roots of average variance extracted (AVE) calculated for each of the constructs are arranged diagonally.

**Table 4:** Goodness of fit.

Constructs/Model	Χ^2^/d.f.	*p*-Value	GFI	CFI	NFI	TLI	RMSEA
Thresholds	<3	>0.05	>0.90	>0.90	>0.90	>0.90	<0.08
Hypothesized Model	2.88	000	0.91	0.94	0.92	0.93	0.07

Note: n = 247, χ^2^/d.f. = 500.679/174.

**Table 5:** Confirmatory factor analysis (CFA) results.

Construct	Item	Factor Loadings	CR	AVE	√AVE	Cronbach’s α
Internal environmental orientation (IEO)	IEO1	0.88 ***	0.94	0.80	0.89	0.94
	IEO2	0.90 ***				
	IEO3	0.91 ***				
	IEO4	0.88 ***				
External environmental orientation (EEO)	EEO5	0.75 ***	0.89	0.66	0.81	0.88
	EEO6	0.90 ***				
	EEO7	0.87 ***				
	EEO8	0.72 ***				
Environmental selection of suppliers (ESS)	ESS9	0.92 ***	0.93	0.82	0.91	0.93
	ESS10	0.93 ***				
	ESS11	0.87 ***				
Environmental monitoring of suppliers (EMS)	EMS12	0.83 ***	0.93	0.81	0.90	0.92
	EMS13	0.94 ***				
	EMS14	0.93 ***				
Environmental collaboration with suppliers (ECS)	ECS15	0.94 ***	0.96	0.94	0.97	0.96
	ECS16	0.96 ***				
	ECS17	0.92 ***				
Firm performance (FP)	ATROI18	0.89 ***	0.94	0.81	0.90	0.94
	EG19	0.96 ***				
	SG20	0.85 ***				
	MSC21	0.89 ***				

Note: n = 247, *** *p* < 0.001, ATROI = After tax returns on investment, EG = Earnings growth, SG = Sales growth, MSC = Market share change.

**Table 6:** Summary results of mediation analysis.

Hypothesis	Path	Direct (c’)	Indirect (ab)	Total (c)	Results
H6a	EEO→ESS→PER	0.048 **	0.199 **	0.247 **	Supported
H6b	EEO→EMS→PER	0.055 **	0.219 **	0.274 **	Supported
H6c	EEO→ECS→PER	0.065 **	0.237 **	0.302 **	Supported
H6d	IEO→ESS→PER	0.056 **	0.356 **	0.412 **	Supported
H6e	IEO→EMS→PER	0.059 **	0.367 ***	0.426 **	Supported
H6f	IEO→ECS→PER	0.064 **	0.407 ***	0.471 **	Supported

Note: ** *p* < 0.01, *** *p* < 0.001, EEO = external environmental orientation, IEO = internal environmental orientation, ESS = environmental selection of suppliers, EMS = environmental monitoring of suppliers, ECS = environmental collaboration with suppliers, PER = firm performance.

**Table 7:** Summary of the results of hypotheses testing.

No.	Hypothesis	Result
H1a	External environmental orientation positively influences environmental selection of suppliers.	supported
H1b	External environmental orientation positively influences environmental monitoring of suppliers.	supported
H1c	External environmental orientation positively influences environmental collaboration with suppliers.	supported
H2a	Internal environmental orientation positively influences environmental selection of suppliers.	supported
H2b	Internal environmental orientation positively influences environmental monitoring of suppliers.	supported
H2c	Internal environmental orientation positively influences environmental collaboration with suppliers.	supported
H3	Environmental selection of suppliers positively influences firm performance.	supported
H4	Environmental monitoring of suppliers positively influences firm performance.	supported
H5	Environmental collaboration with suppliers positively influences firm performance.	supported
H6a	Environmental selection of suppliers mediates the relationship between external environmental orientation and firm performance.	supported
H6b	Environmental monitoring of suppliers mediates the relationship between external environmental orientation and firm performance.	supported
H6c	Environmental collaboration with suppliers mediates the relationship between external environmental orientation and firm performance.	supported
H6d	Environmental selection of suppliers mediates the relationship between internal environmental orientation and firm performance.	supported
H6e	Environmental monitoring of suppliers mediates the relationship between internal environmental orientation and firm performance.	supported
H6f	Environmental collaboration with suppliers mediates the relationship between internal environmental orientation and firm performance.	supported
